# The Effect of Video Modeling on Gymnastics-Based Motor Skills in Children with Autism Spectrum Disorder

**DOI:** 10.3390/healthcare14081009

**Published:** 2026-04-11

**Authors:** Hüseyin Gazi Sönmez, Murat Ergin, Çalık Veli Koçak, Berkan Bozdağ, Ömer Kılınç, Ebru Turan, Umut Canlı, Monira I. Aldhahi

**Affiliations:** 1 Faculty of Humanities and Social Sciences, Abdullah Gül University, Kayseri 38080, Türkiye; dr.hgsonmez@gmail.com; 2 Department of Coaching Education, Aksaray University, Aksaray 68200, Türkiye; muratergin@aksaray.edu.tr; 3 Department of Physical Education and Sport, Aksaray University, Aksaray 68200, Türkiye; velikocak@aksaray.edu.tr (Ç.V.K.); omrklnc1993@gmail.com (Ö.K.); 4 Department of Sports Management, Kırşehir Ahi Evran University, Kırşehir 40100, Türkiye; brknbozdag@gmail.com; 5 Department of Child and Adolescent Mental Health and Disorders, Aksaray University, Aksaray 68200, Türkiye; ebrusglm55@gmail.com; 6 Faculty of Sport Sciences, Tekirdağ Namık Kemal University, Tekirdağ 59030, Türkiye; ucanli@nku.edu.tr; 7 Department of Rehabilitation Sciences, College of Health and Rehabilitation Sciences, Princess Nourah bint Abdulrahman University, Riyadh 11671, Saudi Arabia

**Keywords:** autism spectrum disorder, video modeling, forward roll, gymnastics

## Abstract

**Background and Objectives**: While the effectiveness of video modeling (VM) in teaching academic, daily living, and social skills to individuals with Autism Spectrum Disorder (ASD) is frequently investigated, studies examining the use of VM in teaching gymnastics-based motor skills are limited. This study aimed to examine the effects of VM on the acquisition and maintenance of a gymnastics-based motor skills in preschool children with ASD. **Methods**: The study employed a multiple-probe method across participants in a single-subject research design. Three preschool children diagnosed with mild ASD participated in this study. Baseline, intervention, and follow-up data were systematically collected and analyzed. Social validity data were obtained through semi-structured interviews with parents and special education teachers. **Results**: The percentage of correct responses increased throughout the VM intervention sessions, and all participants reached the proficiency criterion. Follow-up data collected after the intervention showed that the acquired skill was maintained, and the percentages of correct responses ranged from 80% to 100%. Social validity findings revealed that both teachers and parents perceived VM as an effective and feasible teaching approach for teaching motor skills to children with ASD. **Conclusions**: The research findings demonstrate that VM is an effective and socially valid teaching method for teaching and maintaining gymnastics-based motor skills in preschool children with ASD. These results contribute to the existing literature by demonstrating the applicability of video modeling in the context of gymnastics-based training.

## 1. Introduction

Technological advances have reshaped educational practices across many fields, including special education. In recent years, computer-assisted and technology-supported instructional approaches have increasingly been integrated into learning environments for individuals with special needs. Research suggests that when these approaches are designed to align with visual learning preferences, technology-based instruction can be more effective than traditional one to one teaching for individuals with special needs [1].

Within this context, video modeling (VM) has emerged as a particularly prominent method. VM is an instructional approach that involves showing prerecorded videos in which a target behavior or skill is performed correctly, and it is widely used in practice [2]. It is considered an evidence-based strategy, especially for children with autism spectrum disorder (ASD) [3]. At the core of VM lies observational learning, which is recognized as a critical mechanism for individuals with ASD [2,4]. In a typical application, a peer, sibling, or adult model demonstrates the target behavior in a video, and after watching, the learner is encouraged to imitate what they observed [5].

Their tendency to respond well to visual stimuli and video-based environments further enhances the effectiveness of this approach [6]. VM has also been identified as a promising strategy for teaching motor skills [5]. Indeed, research shows that children with ASD often display more task focused behaviors and acquire a greater range of motor skills when taught through video modeling [3]. This approach appears to be particularly useful in the early stages of learning complex motor skills, where observing a model can provide a clear and structured reference for performance [5].

From a practical standpoint, VM offers several advantages over live modeling. It typically requires less instructional time, reduces costs, and can be implemented with relative ease in both school and home settings [7]. The ability to replay videos multiple times allows learners to reinforce their understanding without adding extra demands to the teaching process. Moreover, children with ASD tend to engage more consistently with video content, which further supports observational learning [8]. Another key advantage is that VM can be implemented with less reliance on specialized expertise, making it a more accessible and sustainable option in many contexts [6].

Physical activity plays an important role in supporting children with ASD. Studies indicate that it contributes positively to behavioral regulation, communication skills, and motor development [9,10]. For this reason, early interventions targeting motor skills particularly during the preschool years are especially valuable. Although ASD is primarily characterized by persistent difficulties in social communication and interaction, along with restricted and repetitive patterns of behavior [11], many children with ASD also experience challenges related to postural control, coordination, motor planning, and imitation [12,13]. These difficulties often emerge in early childhood and are closely linked to cognitive, social, and emotional development [14]. Engagement in physical activities can help address these challenges. Gymnastics, in particular, offers substantial benefits for children with ASD by promoting strength, balance, coordination, flexibility, and body control. Beyond physical development, it also supports concentration, self-confidence, and body awareness, while providing a structured and engaging learning environment [15].

A forward roll, although seemingly simple, is a good example of motor learning in practice. This skill has a clear beginning and end and unfolds as a continuous movement, yet it consists of several sequential steps. Because it is performed in a stable environment such as on a mat it is classified as a “closed skill.” Since environmental conditions remain largely unchanged during execution, the individual relies on previously learned and automated motor programs [16]. At the same time, it is a serial skill, involving a sequence of coordinated actions such as keeping the feet together, raising the arms, and placing the hands on the ground. Learning this movement contributes to the development of fundamental motor abilities, including balance, coordination, and body awareness [16,17,18]. Evidence also suggests that children who establish a solid foundation in closed skills at an early age are better equipped to develop decision making and problem solving abilities in more dynamic, open skills later on [19]. In this sense, the forward roll can be viewed as a classic example of motor learning [18,20].

VM has been used in teaching both social and motor skills in early childhood for individuals with ASD [6,21], in the development of motor skills in early growth periods for social interaction, play and daily living [13,22,23], and in sensory integration therapy [24]. In addition, there are studies highlighting the widespread use of single-subject experimental designs in intervention studies aimed at teaching motor skills to children with ASD [22,24].

The literature has extensively examined the effects of video modeling on learning complex skills across a range of domains, including play, aquatic sports, social interaction, rhythmic gymnastics, football, tennis, and golf [6,25,26,27,28,29,30]. However, there is still a notable lack of research focusing on the use of video modeling for fundamental gymnastics skills such as the forward roll.

The forward roll was selected for this study because it is developmentally appropriate for preschool children, relatively easy to implement, and represents a foundational gymnastics skill. At the same time, it is more than just a technical movement; it provides a holistic framework that simultaneously supports balance, core stability, motor planning, and overall motor development [31,32]. In this context, the present study aims to examine the effects of video modeling on the acquisition of the forward roll skill and to contribute to the broader literature on motor skill development.

## 2. Materials and Methods

### 2.1. Research Design

The study employed a multiple-probe design across participants and a single-subject design to examine the effectiveness of the VM on motor skill acquisition. Single-subject research is defined as studies in which the effectiveness of an intervention is evaluated within each subject by making repeated measurements under standard conditions [33]. In the multiple-probe design across participants, the same target behavior is sequentially introduced to different participants in the same setting to demonstrate experimental control. The independent variable was the instruction delivered via VM, and the dependent variable was the acquisition of a motor skill (forward roll) by children with ASD.

### 2.2. Participants

The participants were three preschool children diagnosed with mild ASD (two males and one female) enrolled in a special education kindergarten. Participant selection was conducted in collaboration with school administrators and special education teachers based on predefined inclusion criteria. Although an additional child was initially recruited to account for potential withdrawals, data analyses were conducted with the three participants who met all the criteria and completed the study. Since all three students completed the study, additional student was not needed.

The inclusion criteria required that participants: (a) have a formal diagnosis of mild ASD, (b) be between 5 and 6 years of age, (c) have parental consent, (d) demonstrate the ability to follow modeled actions, (e) attend at least 90% of sessions, (f) comprehend two-step verbal instructions, (g) sustain attention to an activity for at least three minutes, (h) attend to a video presentation for a minimum of 40 s, (i) imitate fine and gross motor movements, (j) fail to perform a forward roll at baseline, and (k) demonstrate basic imitation skills.

All participants were diagnosed with ASD by a child psychiatrist according to DSM-5 criteria, and symptom severity was further characterized using the Childhood Autism Rating Scale (CARS). Participants with CARS scores within the mild range were included in the study; the scores of the three participants were 30.5, 34.5, and 33.5. Students who met the research prerequisites and agreed to participate were given code names instead of their real names (Table 1).

Participant 1 (P1) was a five-year-old male who had attended a special education preschool for 2 years and received 3 h of weekly educational support. He was diagnosed with autism at the age of two. He provides meaningful answers to questions about his daily routines, makes eye contact, and demonstrates basic skills of imitation. He was classified as having mild ASD based on the Childhood Autism Rating Scale (CARS score: 30.5).

Participant 2 (P2) is a five-and-a-half-year-old male who has been attending a special education preschool for two years now. He also received three hours of educational support per week at a private rehabilitation center. He was diagnosed with autism at the age of two. He makes eye contact, demonstrates basic imitation skills, follows instructions consisting of a maximum of two words, communicates using only one word at a time, and does not answer questions. According to the CARS, he has mild ASD (scale score 34.5).

Participant 3 (P3) is a five-year-old female who has been attending a special education preschool for a year. She was diagnosed with autism at the age of one and received three hours of educational support per week at a special rehabilitation center. She makes eye contact, follows and executes instructions consisting of at least two or more words, and demonstrates basic skills. She spoke and expressed herself using single words and made eye contact with the interviewer. According to the CARS, she had mild ASD (scale score of 33.5).

Implementers and Observers: The research team comprised specialists in physical education and sports for individuals with special needs, including academics who have published numerous articles and theses on the motor development and physical activity of students with special needs and who teach courses in the Master’s program in Physical Education and Sport for Individuals with Disabilities. Two researchers held Level 3 Senior Coaching certificates in artistic gymnastics, and one researcher was a child and adolescent psychiatrist. Two observers, a physical education teacher and a graduate student in sports sciences, participated in all sessions to assess the procedural fidelity and interrater reliability.

### 2.3. Task Analyses

Task analysis is the process of breaking down a complex skill or set of behaviors into smaller, teachable units; it also refers to the outcomes of that process [34]. According to Chow and Knudson [35], the better the task objectives and the factors influencing the technique are defined, the easier it will be to intervene in the analysis and implementation. The application of task analysis and the guidance reduction method in behavior modification has been demonstrated in numerous studies. In this study, the task analysis was prepared according to the following scientific principles to ensure its comprehensiveness [34]:

1. Observable and measurable behavioral definitions: The first and second authors developed the task analyses for forward roll together and collaboratively. The researchers broke down the forward roll skill into steps by practicing it themselves. Both authors are senior (International Gymnastics Federation-FIG Academy Level 2) gymnastics instructors. The steps of the forward roll skill were determined by considering the basic motor control, balance, core muscle activation, and body coordination processes described in the literature. For each step, clear observation criteria and success measures were defined to ensure accurate observation of the behavior; For example, specific components such as standing side-by-side are clearly indicated.
2. One-to-one matching with video modeling: The behaviors presented in the video modeling were ensured to be consistent with the steps in the task analysis.
3. Data-driven revision: Performance data obtained during the application were used in a pilot study to check whether the task analysis contained missing or extra components.

The forward roll skill consisted of ten observable sequential steps. The steps are given in Table 2. Each step was independently assessed during the data collection.

### 2.4. Setting and Materials

Video Model Preparation: The VM featured a six-year-old, typically developing, female elite gymnast with national-level competitive experience. The video recordings were conducted in a gymnastics facility and completed in one session. The forward roll skill was recorded based on a predefined task analysis. The duration of the video was 12 s, and the model’s full body was clearly visible throughout the recording [36]. The skill was performed at a natural speed to enhance observational learning [37].

#### Implementation Environment

All pilot and instructional sessions were conducted in the multipurpose hall of a special education kindergarten. The environment was arranged for one-to-one instruction and equipped with gymnastic mats to ensure participant safety. Video instructions were delivered using a tablet device (iPad Air-MCA64TU/A model; Apple Inc, Cupertino, CA, USA), and the participants’ performance was recorded using a digital video camera (Canon EOS 2000D model; Canon Inc, Tokyo, Japan). This camera has a 24.7-megapixel, 1080p full HD resolution and records videos at 3 fps.

Pilot applications and training trials were conducted in the multipurpose hall of the special education kindergarten. The learning environment had a ceiling approximately 4 m high and measured 7 × 12 m in size. The learning environment was arranged to be suitable for one-on-one instruction. The floor of the learning environment was covered with a soft material (tatami). The video images were shown to the students at a table 5 m away from the gymnastics mat.

### 2.5. Procedure

The study comprised four phases: pilot application, baseline, intervention, and follow-up.

#### 2.5.1. Pilot Application

A pilot study was conducted with a preschool child diagnosed with mild ASD who did not participate in the main study. Based on the observations of performance and comprehension, minor revisions were made to the VM to improve clarity.

#### 2.5.2. Baseline Phase

The stages of the multiple baseline models among participants are as follows: (a) Target behaviors to be acquired by each participant are determined and defined. (b) Data is collected by continuously observing all three participants during the baseline phase. (c) After obtaining stable data from the first participant, the independent variable is applied. During this time, baseline data collection continues for the other participants. (d) Application continues until the criterion is met and stable data is obtained from the first participant. (e) Application begins with the second participant, but baseline data is still being collected from the third participant. (f) Application begins with the second participant and continues until stable data is obtained. (g) Application continues with the third participant until the criterion is met and stable data is obtained [38].

The training environment was arranged by the researchers. After the environment was arranged, the students were brought into the training environment one by one by the researchers, and each student was allowed to examine the gymnastic equipment for one minute. Baseline data were collected independently for each participant until stable performance was observed for at least three consecutive sessions.

The practitioner said to the participant, “Today we’ll be doing an exercise together, would you like to work with me?” Positive responses were reinforced with “well done.” The practitioner then instructed the participant to “roll forward,” and waited five seconds for the participant’s response. Steps correctly performed were marked with a “+” on the Data Recording Form. Steps not performed or not responded to were considered incorrect and marked with a “-”. The initial data was repeated for all three participants until at least three stable data points were obtained consecutively. The baseline levels of the three participants were 0%.

#### 2.5.3. Intervention Phase

The intervention sequence for participants is based on a multiple baseline across participants design. In this design, each participant’s initiation of the intervention depends on the previous participant meeting the 80% success criterion set in the baseline phase. The accuracy values required for a skill to be considered learned range from 80% to 100%. The criterion of 80% is often used as the lower limit of this range and indicates that the performance is not accidental [34,39,40,41]. Furthermore, when success is sustained over several consecutive sessions, it can be inferred that the learned process is permanent and generalizable [42,43].

The study was conducted over eight weeks, two days per week, with one session per day and three trials per session (30-40 s). To prepare the body for performing the forward roll in gymnastics, a total of five minutes of warm-up exercises were performed, including two minutes of slow-paced running, general warm-up exercises, and gymnastics-specific warm-up exercises.

During the video viewing phase, the practitioner and participant sat side-by-side, and the practitioner asked, “We’re going to watch a video together, would you like to?” Positive responses were reinforced with “Well done.” The practitioner demonstrated the reinforcement by saying, “If you sit on the chair with your hands on the table and your feet on the floor, you can play with this car after watching the video.”

“The video footage was shown to Participant for a second time. The skill steps were paused while watching the video to draw attention to each step. He was reinforced with, “Well done, you’re watching very carefully.” The videos of the other steps were shown to Participant in a similar manner, and he completed watching the video footage.

Participant’s sitting still and watching the video was reinforced with, “Well done, you worked very well.” Then, saying, “Now, let’s roll forward like your friend in the video,” they went to the head of the gymnastics mat. The practitioner gave the instruction, “roll forward.” Participant’s response was waited for five seconds. Skill steps he performed correctly were marked with a “+” on the Data Record Form, and skill steps he performed incorrectly or showed no response to were marked with a “-.” The steps Participant makes incorrectly are ignored, and he is instructed to move on to the next step. The next session is repeated using a video application, reinforcing the students’ behaviour of participating in the skill. Instruction continues until all skill steps are completed, and after three attempts in that session, reinforcement is given by saying, “Well done, you worked very well, now you can play with the car.” Students are reinforced with a continuous reinforcement schedule. The instructors gave the participants no clues.

#### 2.5.4. Follow-Up Phases

Follow-up sessions were conducted in the first, second, and third weeks post intervention to assess the maintenance of the acquired skill. The procedures during the probe and follow-up sessions were identical to those during the baseline.

### 2.6. Data Collection and Analysis

The data obtained were evaluated by two expert academics in the field of Physical Education and Sports for Individuals with special needs. The data were recorded in Excel and analyzed using a line graph, which is a graphical analysis method. The data from the data recording form, including students’ baseline, application, attendance, and follow-up data, the number of correct response percentages, and the total number of steps in the skill analysis, were divided by 100 to find the percentage of correct response percentages on the vertical axis, which was then plotted on the graph.

#### 2.6.1. Interobserver Reliability and Procedural Fidelity

An inter-observer reliability study was conducted by two independent observers who watched video recordings of 30% of the randomly selected sessions of the experiment and used the “Application Reliability Form” to evaluate whether the researchers exhibited the required behaviors at each stage of the experiment. The reliability coefficient was determined using the formula “[(agreement)/(agreement + disagreement)] × 100” [44]. The interobserver reliability was 100% across all sessions. Researchers determined whether the independent variable was implemented as planned by conducting procedural fidelity studies [45]. For this purpose, two independent observers watched video recordings of 30% of randomly selected sessions of the experiment and assessed whether the researchers exhibited the required behaviors at each stage of the experiment using an “Application Reliability Form”.

The responses were then analyzed using the formula “[(observed practitioner behavior/planned practitioner behavior) × 100)]” and the procedural fidelity coefficient was obtained [46]. The procedural fidelity coefficient of the participants in the experiment was >90%.

#### 2.6.2. Social Validity

Social validity data were collected through semi-structured interviews with three special education teachers and three parents. For this purpose, the first author, drawing on previous video-based modeling research [6,47,48], developed two questionnaires to assess social validity after the completion of the study. The teacher form consisted of four open-ended questions, and the parent form consisted of three open-ended questions. Upon completion of the research process, the participants’ families and teachers were shown video footage of the baseline sessions, teaching sessions, and follow-up sessions conducted in the study, and then asked to answer the questions in the social validity forms. After obtaining the participants’ permission, audio recordings were made, and the interviews lasted approximately 10–20 min. The interviews were conducted face-to-face in the Guidance Counselor’s office. The responses were recorded and analyzed descriptively. Data reliability was ensured through independent expert review and consistency checks. Teachers were asked about (a) whether teaching with VM was beneficial, (b) their positive and negative opinions about VM, (c) whether teaching with VM could be used in teaching other motor skills, and (d) the benefits it provided to students. Parents were asked about (a) their positive and negative opinions about VM, (b) whether VM could be used in teaching other motor skills, and (c) the benefits it provided to students.

## 3. Results

**Figure 1:**
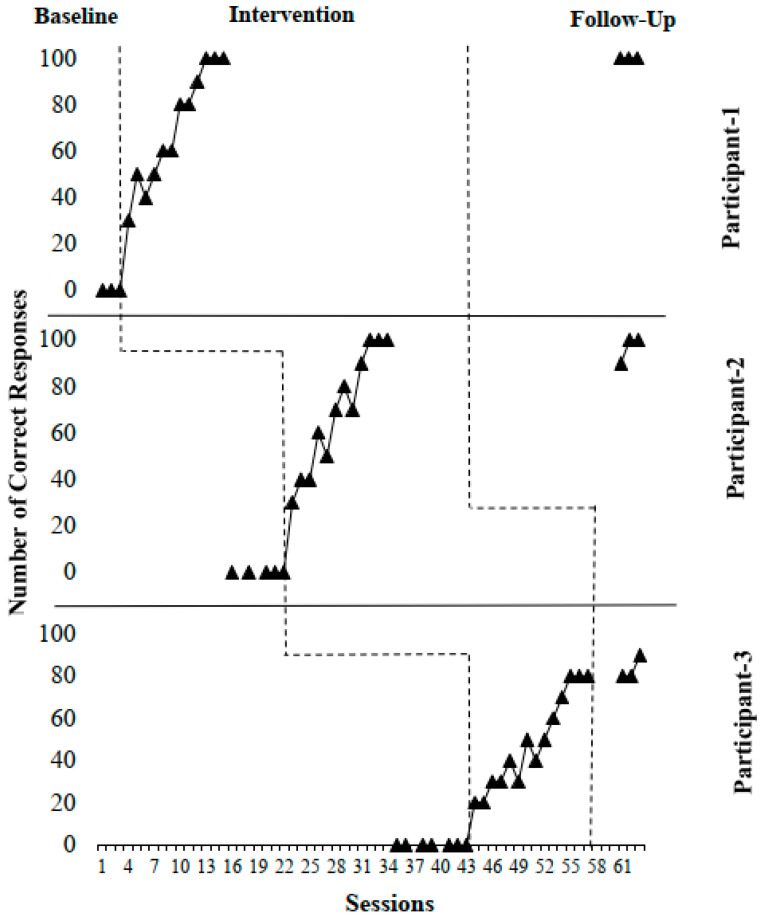
Correct response percentages regarding forward roll skills.

The percentages of correct answers regarding the participants’ advanced forward roll skills are shown in Figure 1.

In the study, it was determined that the post-intervention data was 100% for Participants 1 and 2, and 80% for Participant 3 (Table 3).

### 3.1. Effectiveness Findings

Figure 1 presents the results of teaching forward roll skills with VM, revealing an overall increase in the number of correct answers in all three students. All participants initially showed 0% correct answers. Twelve practice sessions were conducted for participant 1. A rapid increase was observed from the first session, reaching the 80% threshold from the seventh session onwards, and stable data was obtained. The percentages of correct answers in the practice sessions were calculated as 30%, 50%, 40%, 50%, 60%, 60%, 80%, 80%, 90%, 100%, 100%, and 100%, respectively.

Twelve practice sessions were conducted for participant 2. The percentages of correct answers in the application sessions were calculated as 30%, 40%, 40%, 60%, 50%, 70%, 80%, 70%, 90%, 100%, 100%, and 100%, respectively.

14 application sessions were conducted for the 3rd participant. After the twelfth session, it was observed that the 80% criterion was met and stable data was obtained. The percentages of correct answers in the application sessions were calculated as 20%, 20%, 30%, 30%, 40%, 30%, 50%, 40%, 50%, 60%, 70%, 80%, 80%, and 80%, respectively.

The application phase was terminated after three participants achieved the target skill with stable data in three consecutive sessions. When the data obtained before and after the application were compared, it was seen that all 3 participants acquired the target skill.

Participant 1 achieved an 80% correct answer rate by the end of session 12 and a 100% correct answer rate by the end of session 13. Participant 2 achieved an 80% correct answer rate by the end of session 14 and a 100% correct answer rate by the end of session 15. Participant 3 achieved an 80% correct answer rate by the end of session 19.

### 3.2. Follow-Up Findings

Examining the follow-up data in Figure 1, Participant 1 achieved 100%, 100%, and 100% correct answer rates in the first, second, and third weeks after the intervention, respectively; Participant 2 achieved 90%, 100%, and 100% correct answer rates; and Participant 3 achieved 80%, 80%, and 90% correct answer rates. These findings demonstrate that participants were able to maintain the forward roll skill they acquired through video modeling.

Participant 1 maintained their gains with a 100% correct answer rate in all 3 follow-up sessions. Participant 2 reached a 90% correct answer rate at the end of session 18, and a 100% correct answer rate at the end of sessions 19 and 20. Participant 3 reached an 80% correct answer rate at the end of sessions 22 and 23, and a 90% correct answer rate at the end of session 24.

### 3.3. Social Validity Findings

All teachers stated that VM was beneficial, that no negative findings were observed in the study, and that it could be used to teach complex skills, such as gymnastics, to students with ASD. Furthermore, all teachers indicated that VM could be easily used outside school by parents and other professionals and that students with ASD are more receptive to technology and visual stimuli in their learning. Researchers observed that students enjoyed participating in VM instructional activities, enjoyed watching the video of the forward roll skill, and wanted to learn other movements by watching the videos. All students’ parents stated that the VM was beneficial and could be used to teach other movement skills, besides the forward roll. Two teachers reported that their students gained self-confidence, while one teacher and one parent reported that it positively contributed to the students’ development through increased motivation and enjoyment of the activity.

## 4. Discussion

The purpose of the present study was to examine the effects of VM on the acquisition and maintenance of gymnastics-based motor skills in preschool children with ASD. The findings indicate that the VM effectively improved the correct performance of the forward roll for all participants. All participants met the mastery criterion [34,39,40,41,49] of at least an 80% correct response rate for the forward roll skill. Furthermore, these performance gains were maintained during follow-up sessions conducted for up to three weeks after the intervention.

The current study findings show that video modeling training can be effective not only in teaching simple skills but also complex skills. While the literature generally focuses on imitation games and daily living skills with video modeling training, this study is considered to contribute to the existing literature by aiming to teach gymnastics skills in preschool children with ASD [50,51,52,53].

To date, no study has directly examined the effectiveness of VM in teaching forward roll skills to preschool children with ASD. Therefore, the findings of the present study were compared with those of other indirectly related studies. For example, Phillips [54] reported that a behavioral coaching intervention incorporating VM, video feedback, and verbal feedback was more effective than VM alone in teaching Taekwondo strikes to individuals with ASD. In contrast, the current findings demonstrate that VM alone can be sufficient to facilitate motor skill acquisition when applied to fundamental gymnastic skills. These results contribute to the existing literature by demonstrating the applicability of video modeling in the context of gymnastics-based training.

The observed improvements following the introduction of VM are consistent with previous research demonstrating the effectiveness of video-based instruction in teaching motor-related skills to children with ASD [4,26]. Although VM is often implemented alongside additional feedback strategies, particularly in studies involving typically developing individuals [55], the present study provides evidence that VM alone can support motor skill learning in young children with ASD.

Similarly, Baudry et al. [56] suggested that the VM supports the development of a cognitive representation of movement and found that the VM was effective in teaching and maintaining a complex gymnastics skill (double-leg circle on the pommel horse). Together, these findings support the notion that the VM facilitates motor learning by enhancing observational learning and motor planning processes.

Beyond motor performance outcomes, positive social validity findings further emphasize the practical value of the VM training. Teachers and parents emphasized that VM was easy to implement, engaging for children, and adaptable to educational and home settings. These perceptions are consistent with previous research reporting the high acceptability and feasibility of VM interventions [51,52].

Gymnastics offers a structured and adaptable context for developing fundamental motor skills, and improvements in motor performance may positively contribute to self-confidence and social participation in children. Given the documented benefits of gymnastics for balance, coordination, and motor planning in children with ASD [57], teaching fundamental gymnastics skills through a VM may support the holistic development of this population.

Overall, the present findings, together with similar outcomes reported in the literature, contribute to a growing body of evidence supporting VM as an effective and socially valid instructional approach for children with ASD. By demonstrating its application to a fundamental gymnastics’ skill, the findings highlight the potential of the VM as a practical tool for promoting motor development within special education and physical activity contexts.

## 5. Conclusions

The findings of this study indicate that VM is an effective and socially valid instructional method for teaching and maintaining the forward roll skill in preschool children with ASD. The results contribute to the limited literature in this field supporting the use of video modeling in gymnastics-based motor skills training. Video modeling represents a practical, accessible, and engaging teaching approach for educators and families of young children with ASD.

### 5.1. Strengths of the Study

One of the strengths of this study is that all three participants achieved a master skill level in performing forward roll and maintained this skill in follow-up sessions after the intervention. Second, the key requirements for demonstrating experimental control and internal validity in a single-subject research design are maintaining a stable baseline in the follow-up phase, changing only a single variable, and observing a consistent change in data collection over time.

### 5.2. Limitations and Recommendations of the Study

This study had several limitations that should be considered. First, the investigation focused on a single motor skill and included a small number of participants, which is a characteristic of single-subject research design. Second, the generalization of the acquired skills to other settings or skills has not been assessed. Future research should examine the effects of VM on a broader range of gymnastics and sport-specific motor skills, including generalization and social participation measures, and explore its effectiveness across different age groups and disability profiles. Larger-scale studies may further strengthen the evidence base for VM-based motor skill training. Despite the consistency of improvements observed in the current research, the findings should be interpreted cautiously due to the single-subject design and limited sample size. Therefore, the conclusions are restricted to the specific participants, skills, and instructional contexts examined in this study.

## Figures and Tables

**Table 1:** Demographic information of participants.

Participation	Sex	Ages	Primary Exceptionality	Kindergarten Attendance Period
P 1	male	five	ASD	two years
P 2	male	five	ASD	two years
P 3	female	five	ASD	two years

**Table 2:** Task analysis of forward roll skill.

Step	Forward Roll Skill Task Analysis
1	standing with feet side by side
2	raising arms.
3	placing hands on a mat in front of feet.
4	bringing head between arms.
5	bringing chin to chest.
6	bringing head to knees.
7	bending knees
8	rolling forward.
9	standing up.
10	raising arms.

**Table 3:** Participant probe-by-probe performance (number of correct steps/total steps).

Skill	Participant	Baseline	Intervention	Follow-Up
Forward roll	P1	0/100	100/100	100/100
	P2	0/100	100/100	100/100
	P3	0/100	80/100	80/100

## Data Availability

The data presented in this study are available from the corresponding author upon request due to participant confidentiality.

## Abbreviations

The following abbreviations are used in this manuscript:

ASD	Autism Spectrum Disorder
VM	Video modeling
CARS	Childhood Autism Rating Scale
